# (+)-{1,2-Bis[(2*R*,5*R*)-2,5-diethyl­phospho­lan-1-yl]ethane-*κ* ^2^ *P*,*P*′}(*η* ^4^-cyclo­octa-1,5-diene)rhodium(I) tetra­fluoridoborate

**DOI:** 10.1107/S1600536810039577

**Published:** 2010-10-09

**Authors:** Stefan Schulz, Christian Fischer, Hans-Joachim Drexler, Detlef Heller

**Affiliations:** a Leibniz-Institut für Katalyse e. V. an der Universität Rostock, Albert-Einstein-Str. 29a, 18059 Rostock, Germany

## Abstract

The title compound, [Rh(C_8_H_12_)(C_18_H_36_P_2_)]BF_4_, exhibits a rhodium(I) complex cation with a bidentate bis­phosphine ligand and a bidentate *η* ^2^,*η* ^2^-coordinated cyclo­octa-1,5-diene ligand. The ligands form a slightly distorted square-planar coordination environment for the Rh(I) atom. An intra­molecular P–Rh–P bite angle of 83.91 (2)° is observed. The dihedral angle between the P—Rh—P and the *X*—Rh—*X* planes (*X* is the centroid of a double bond) is 14.0 (1)°. The BF_4_ anion is disordered over two positions in a 0.515 (7):0.485 (7) ratio.

## Related literature

For general synthetic aspects and different related structures of cationic rhodium bis­phosphine diolefin complexes, see: Schulz *et al.* (2010 ▶) and references cited therein. For applications of the Et-BPE ligand {Et-BPE (1,2-bis[(2*R*,5*R*)-2,5-diethylphospholan-1-yl]ethane)} in catalytic reactions, see: Axtell *et al.* (2005 ▶); Jerphagnon *et al.* (2003 ▶); Burk *et al.* (1998 ▶). For related structures, see: Burk *et al.* (1990 ▶); Drexler *et al.* (2001 ▶, 2004 ▶).
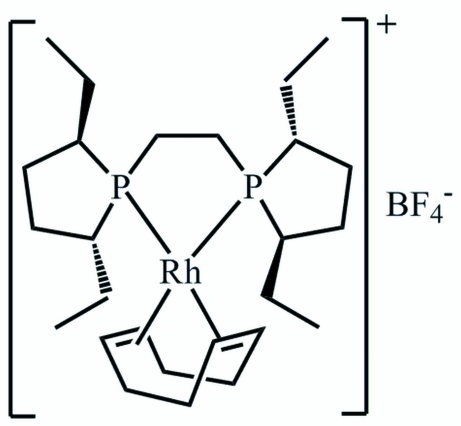


## Experimental

### 

#### Crystal data

[Rh(C_8_H_12_)(C_18_H_36_P_2_)]BF_4_
*M* *_r_* = 612.32
Orthorhombic, 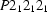

*a* = 8.8374 (18) Å
*b* = 16.218 (3) Å
*c* = 19.946 (4) Å
*V* = 2858.7 (10) Å^3^
*Z* = 4
Mo *K*α radiation
μ = 0.75 mm^−1^
*T* = 200 K
0.50 × 0.43 × 0.40 mm

#### Data collection

STOE IPDS 2 diffractometer
Absorption correction: numerical (*X-SHAPE*; Stoe & Cie, 2005) *T*_min_ = 0.728, *T*_max_ = 0.858
36650 measured reflections
6075 independent reflections
5779 reflections with *I* > 2σ(*I*)
*R*_int_ = 0.035

#### Refinement

*R*[*F* ^2^ > 2σ(*F* ^2^)] = 0.027
*wR*(*F* ^2^) = 0.073
*S* = 1.06
6075 reflections
305 parameters
21 restraints
H-atom parameters constrained
Δρ_max_ = 0.95 e Å^−3^
Δρ_min_ = −0.34 e Å^−3^
Absolute structure: Flack (1983 ▶), 2621 Friedel pairs
Flack parameter: −0.02 (2)

### 

Data collection: *X-AREA* (Stoe & Cie, 2005) ▶; cell refinement: *X-AREA* ▶; data reduction: *X-RED32* (Stoe & Cie, 2005) ▶; program(s) used to solve structure: *SHELXS97* (Sheldrick, 2008 ▶); program(s) used to refine structure: *SHELXL97* (Sheldrick, 2008 ▶); molecular graphics: *SHELXTL* (Sheldrick, 2008 ▶); software used to prepare material for publication: *SHELXTL*.

## Supplementary Material

Crystal structure: contains datablocks I, global. DOI: 10.1107/S1600536810039577/si2281sup1.cif

Structure factors: contains datablocks I. DOI: 10.1107/S1600536810039577/si2281Isup2.hkl

Additional supplementary materials:  crystallographic information; 3D view; checkCIF report

## Figures and Tables

**Fig. 1.**
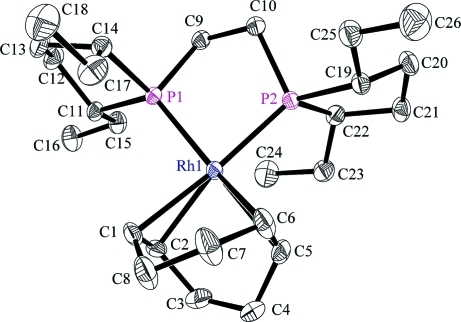
Perspective view and numbering scheme of the cation [Rh((R,R)-Et-BPE)COD]+. All H atoms and the anion have been omitted for clarity. Thermal ellipsoids are drawn at the 30% probability level.

## supplementary crystallographic information

### Comment

The geometry of the cation of the title compound is comparable with the three independent complexes described in Schulz *et al.* (2010): the dihedral angle between the planes P1,Rh1,P2 and X1,Rh1,X2 (*X* = centroid of the double bond) of 14.0 (1)° corresponds to those in the Me-BPE complexes (14.7 (1)°, 14.8 (1)° and 15.3 (1)°). Furthermore, the dihedral angle is not the value found for the corresponding Et-DuPhos complex [Rh((*R*,*R*)-Et-DuPhos)COD]BF_4_ (21.1 (1)°) (Drexler *et al.*, 2001) or for the complex [Rh((*R*,*R*)-Me-BPE)COD]SbF_6_ (19.4°) (Burk *et al.*, 1990; Drexler *et al.*, 2004). The cationic rhodium bisphosphine diolefine complexes build up five-membered ring chelates with rhodium (Fig. 1). For the title compound the molecular structure shows a *δ*-conformation of the backbone of the bisphosphine. The COD is *η*^2^,*η*^2^-coordinated and is orientated in an anticlockwise twist manner. An intramolecular P1—Rh1—P2 angle of 83.91 (2)° is obtained. This is in the same range of corresponding complexes already described in the literature Schulz *et al.* (2010) and literature therein. The bonds Rh1—P1 and Rh1—P2 show bond lengths of 2.2754 (8) Å and 2.2702 (8) Å, respectively. Applications of the ligand Et-BPE in catalytic reactions are reported by Axtell *et al.* (2005); Jerphagnon *et al.* (2003) and Burk *et al.* (1998).

### Experimental

By overlaying a solution of [Rh((*R*,*R*)-Et-BPE)COD)]BF_4_ in dichloromethane with MTBE (methyl-*tert*-butylether) red single crystals suitable for X-ray analysis are obtained. ^31^P NMR (CD_2_Cl_2_, 298 K, 162 MHz) [p.p.m.]: 73.5 (d, *J*_P—Rh_ = 145.5 Hz).

### Refinement

All non-hydrogen atoms are refined anisotropically, except not fully occupied fluorine atoms of the anion. All H atoms were placed in idealized positions with d(C—H) = 0.98(CH), 0.97 (CH_2_) and 0.96 (CH_3_) Å and refined using a riding model with *U*_iso_(H) fixed at 1.5 *U*_eq_(C) for CH_3_ and 1.2 *U*_eq_(C) for CH_2_ and CH. The absolute configuration indicators 2*R*,5*R* for the title compound were determined by using 2621 Friedel pairs in the refinement. The Flack parameter at convergence was -0.02 (2).

### Crystal data

**Table:**

[Rh(C_8_H_12_)(C_18_H_36_P_2_)]BF_4_	*F*(000) = 1280
*M**_r_* = 612.32	*D*_x_ = 1.423 Mg m^−^^3^
Orthorhombic, *P*2_1_2_1_2_1_	Mo *K*α radiation, λ = 0.71073 Å
Hall symbol: P 2ac 2ab	Cell parameters from 54003 reflections
*a* = 8.8374 (18) Å	θ = 1.6–27.2°
*b* = 16.218 (3) Å	µ = 0.75 mm^−^^1^
*c* = 19.946 (4) Å	*T* = 200 K
*V* = 2858.7 (10) Å^3^	Part of block, red
*Z* = 4	0.50 × 0.43 × 0.40 mm

### Data collection

**Table:**

STOE IPDS 2 diffractometer	6075 independent reflections
Radiation source: fine-focus sealed tube	5779 reflections with *I* > 2σ(*I*)
graphite	*R*_int_ = 0.035
Detector resolution: 6.67 pixels mm^-1^	θ_max_ = 26.8°, θ_min_ = 1.6°
rotation method scans	*h* = −11→11
Absorption correction: numerical (*X-SHAPE*; Stoe & Cie, 2005)	*k* = −20→20
*T*_min_ = 0.728, *T*_max_ = 0.858	*l* = −25→25
36650 measured reflections	

### Refinement

**Table:**

Refinement on *F*^2^	Secondary atom site location: difference Fourier map
Least-squares matrix: full	Hydrogen site location: inferred from neighbouring sites
*R*[*F*^2^ > 2σ(*F*^2^)] = 0.027	H-atom parameters constrained
*wR*(*F*^2^) = 0.073	*w* = 1/[σ^2^(*F*_o_^2^) + (0.0545*P*)^2^ + 0.2291*P*] where *P* = (*F*_o_^2^ + 2*F*_c_^2^)/3
*S* = 1.06	(Δ/σ)_max_ = 0.002
6075 reflections	Δρ_max_ = 0.95 e Å^−^^3^
305 parameters	Δρ_min_ = −0.34 e Å^−^^3^
21 restraints	Absolute structure: Flack (1983), 2621 Friedel pairs
Primary atom site location: structure-invariant direct methods	Flack parameter: −0.02 (2)

### Special details

**Table:**

Geometry. All e.s.d.'s (except the e.s.d. in the dihedral angle between two l.s. planes) are estimated using the full covariance matrix. The cell e.s.d.'s are taken into account individually in the estimation of e.s.d.'s in distances, angles and torsion angles; correlations between e.s.d.'s in cell parameters are only used when they are defined by crystal symmetry. An approximate (isotropic) treatment of cell e.s.d.'s is used for estimating e.s.d.'s involving l.s. planes.
Refinement. Refinement of *F*^2^ against ALL reflections. The weighted *R*-factor *wR* and goodness of fit *S* are based on *F*^2^, conventional *R*-factors *R* are based on *F*, with *F* set to zero for negative *F*^2^. The threshold expression of *F*^2^ > σ(*F*^2^) is used only for calculating *R*-factors(gt) *etc*. and is not relevant to the choice of reflections for refinement. *R*-factors based on *F*^2^ are statistically about twice as large as those based on *F*, and *R*- factors based on ALL data will be even larger.

### Fractional atomic coordinates and isotropic or equivalent isotropic displacement parameters (Å^2^)

**Table:**

	*x*	*y*	*z*	*U*_iso_*/*U*_eq_	Occ. (<1)
Rh1	0.55265 (2)	0.179334 (12)	0.820495 (9)	0.02703 (6)	
P1	0.62151 (8)	0.15079 (4)	0.71305 (3)	0.02599 (13)	
P2	0.62220 (8)	0.04667 (4)	0.83972 (3)	0.02676 (13)	
C1	0.5242 (4)	0.31550 (18)	0.80272 (15)	0.0417 (7)	
H1A	0.5663	0.3348	0.7600	0.050*	
C2	0.3872 (4)	0.2772 (2)	0.79692 (15)	0.0431 (7)	
H2A	0.3502	0.2749	0.7506	0.052*	
C3	0.2629 (5)	0.2709 (3)	0.8481 (2)	0.0656 (11)	
H3A	0.1969	0.2254	0.8362	0.079*	
H3B	0.2032	0.3211	0.8470	0.079*	
C4	0.3214 (6)	0.2576 (3)	0.9195 (2)	0.0721 (13)	
H4A	0.3489	0.3106	0.9384	0.087*	
H4B	0.2401	0.2351	0.9466	0.087*	
C5	0.4564 (6)	0.2006 (2)	0.92417 (14)	0.0550 (10)	
H5A	0.4380	0.1500	0.9499	0.066*	
C6	0.6040 (6)	0.2249 (2)	0.92248 (16)	0.0555 (11)	
H6A	0.6720	0.1888	0.9479	0.067*	
C7	0.6635 (7)	0.3112 (2)	0.91348 (19)	0.0689 (13)	
H7A	0.7685	0.3079	0.8995	0.083*	
H7B	0.6612	0.3389	0.9566	0.083*	
C8	0.5783 (6)	0.3636 (2)	0.86315 (19)	0.0667 (13)	
H8A	0.4916	0.3883	0.8852	0.080*	
H8B	0.6437	0.4080	0.8481	0.080*	
C9	0.6456 (3)	0.03874 (18)	0.70368 (13)	0.0333 (6)	
H9A	0.5478	0.0122	0.6988	0.040*	
H9B	0.7057	0.0267	0.6642	0.040*	
C10	0.7254 (3)	0.00717 (18)	0.76640 (13)	0.0340 (6)	
H10A	0.8294	0.0264	0.7672	0.041*	
H10B	0.7259	−0.0526	0.7668	0.041*	
C11	0.5147 (3)	0.18501 (18)	0.63828 (12)	0.0309 (5)	
H11A	0.4830	0.2421	0.6462	0.037*	
C12	0.6369 (4)	0.1865 (2)	0.58392 (14)	0.0435 (7)	
H12A	0.6605	0.1308	0.5696	0.052*	
H12B	0.6018	0.2174	0.5453	0.052*	
C13	0.7764 (4)	0.2272 (2)	0.61365 (17)	0.0484 (8)	
H13A	0.7610	0.2863	0.6161	0.058*	
H13B	0.8631	0.2168	0.5850	0.058*	
C14	0.8070 (3)	0.19301 (18)	0.68390 (14)	0.0364 (6)	
H14A	0.8773	0.1466	0.6793	0.044*	
C15	0.3730 (4)	0.1352 (2)	0.62165 (15)	0.0414 (7)	
H15A	0.3083	0.1328	0.6609	0.050*	
H15B	0.4020	0.0793	0.6102	0.050*	
C16	0.2847 (4)	0.1727 (3)	0.56339 (17)	0.0522 (8)	
H16A	0.1960	0.1402	0.5549	0.078*	
H16B	0.3473	0.1735	0.5241	0.078*	
H16C	0.2553	0.2280	0.5746	0.078*	
C17	0.8803 (4)	0.2564 (3)	0.7303 (2)	0.0556 (9)	
H17A	0.8119	0.3026	0.7362	0.067*	
H17B	0.8971	0.2316	0.7739	0.067*	
C18	1.0305 (5)	0.2877 (3)	0.7027 (3)	0.0729 (12)	
H18A	1.0730	0.3272	0.7333	0.109*	
H18B	1.0140	0.3135	0.6600	0.109*	
H18C	1.0991	0.2423	0.6975	0.109*	
C19	0.7298 (3)	0.01375 (18)	0.91399 (14)	0.0338 (6)	
H19A	0.6911	0.0451	0.9523	0.041*	
C20	0.6821 (4)	−0.07572 (19)	0.92375 (17)	0.0449 (7)	
H20A	0.7293	−0.1108	0.8903	0.054*	
H20B	0.7111	−0.0951	0.9679	0.054*	
C21	0.5106 (4)	−0.07681 (19)	0.91604 (16)	0.0428 (7)	
H21A	0.4637	−0.0499	0.9542	0.051*	
H21B	0.4747	−0.1333	0.9143	0.051*	
C22	0.4677 (3)	−0.03148 (17)	0.85109 (14)	0.0345 (6)	
H22A	0.4750	−0.0711	0.8141	0.041*	
C23	0.3065 (4)	0.0006 (2)	0.85289 (17)	0.0448 (7)	
H23A	0.3000	0.0436	0.8865	0.054*	
H23B	0.2402	−0.0439	0.8668	0.054*	
C24	0.2487 (4)	0.0349 (3)	0.7865 (2)	0.0622 (10)	
H24A	0.1466	0.0540	0.7920	0.093*	
H24B	0.2513	−0.0076	0.7530	0.093*	
H24C	0.3118	0.0800	0.7727	0.093*	
C25	0.9010 (4)	0.0292 (2)	0.91028 (17)	0.0456 (7)	
H25A	0.9430	−0.0031	0.8738	0.055*	
H25B	0.9186	0.0869	0.9003	0.055*	
C26	0.9818 (5)	0.0075 (4)	0.9735 (2)	0.0775 (13)	
H26A	1.0879	0.0184	0.9683	0.116*	
H26B	0.9669	−0.0499	0.9832	0.116*	
H26C	0.9425	0.0401	1.0097	0.116*	
B1	0.5008 (3)	0.0287 (2)	0.09541 (15)	0.0521 (9)	
F1	0.4824 (5)	0.0657 (3)	0.15617 (16)	0.1419 (18)	
F2	0.4003 (6)	0.0460 (4)	0.0449 (2)	0.0663 (15)*	0.515 (7)
F3	0.6464 (5)	0.0467 (4)	0.0748 (3)	0.0791 (18)*	0.515 (7)
F4	0.4794 (10)	−0.0470 (4)	0.1253 (4)	0.125 (3)*	0.515 (7)
F2'	0.3620 (5)	0.0229 (4)	0.0636 (3)	0.0655 (15)*	0.485 (7)
F3'	0.5818 (10)	0.0895 (5)	0.0629 (4)	0.111 (3)*	0.485 (7)
F4'	0.5614 (7)	−0.0489 (3)	0.0851 (3)	0.0781 (19)*	0.485 (7)

### Atomic displacement parameters (Å^2^)

**Table:**

	*U*^11^	*U*^22^	*U*^33^	*U*^12^	*U*^13^	*U*^23^
Rh1	0.03754 (10)	0.02407 (10)	0.01948 (9)	0.00346 (8)	−0.00143 (7)	−0.00007 (7)
P1	0.0289 (3)	0.0278 (3)	0.0213 (3)	0.0005 (2)	0.0010 (2)	0.0001 (2)
P2	0.0317 (3)	0.0240 (3)	0.0246 (3)	0.0016 (3)	−0.0005 (2)	0.0006 (2)
C1	0.0641 (19)	0.0273 (13)	0.0336 (13)	0.0106 (14)	−0.0043 (12)	0.0059 (11)
C2	0.0526 (17)	0.0443 (17)	0.0324 (13)	0.0261 (15)	0.0063 (13)	0.0056 (12)
C3	0.062 (2)	0.076 (3)	0.059 (2)	0.030 (2)	0.0252 (19)	0.006 (2)
C4	0.107 (4)	0.065 (3)	0.044 (2)	0.028 (2)	0.037 (2)	0.0032 (18)
C5	0.104 (3)	0.0408 (18)	0.0198 (12)	0.0109 (19)	0.0115 (17)	0.0005 (11)
C6	0.111 (3)	0.0291 (16)	0.0265 (14)	0.0103 (18)	−0.0235 (17)	−0.0037 (12)
C7	0.126 (4)	0.0322 (18)	0.0481 (19)	−0.002 (2)	−0.034 (2)	−0.0029 (15)
C8	0.124 (4)	0.0303 (17)	0.0455 (18)	0.002 (2)	−0.015 (2)	0.0045 (14)
C9	0.0405 (15)	0.0334 (14)	0.0260 (12)	0.0011 (11)	0.0036 (10)	−0.0019 (11)
C10	0.0411 (15)	0.0310 (14)	0.0299 (13)	0.0040 (11)	0.0038 (11)	−0.0001 (11)
C11	0.0391 (14)	0.0326 (13)	0.0210 (10)	0.0000 (11)	−0.0007 (9)	0.0014 (10)
C12	0.0479 (17)	0.057 (2)	0.0253 (12)	0.0031 (16)	0.0055 (11)	0.0059 (13)
C13	0.0458 (17)	0.061 (2)	0.0386 (16)	−0.0046 (15)	0.0080 (13)	0.0160 (15)
C14	0.0327 (13)	0.0392 (15)	0.0371 (14)	0.0000 (10)	0.0009 (11)	0.0051 (12)
C15	0.0480 (17)	0.0448 (17)	0.0314 (14)	−0.0022 (14)	−0.0072 (12)	0.0045 (12)
C16	0.0573 (19)	0.057 (2)	0.0426 (16)	−0.0054 (18)	−0.0188 (14)	0.0081 (17)
C17	0.0420 (17)	0.063 (2)	0.062 (2)	−0.0173 (17)	0.0009 (16)	−0.0054 (18)
C18	0.047 (2)	0.085 (3)	0.087 (3)	−0.024 (2)	−0.0018 (19)	0.002 (2)
C19	0.0390 (15)	0.0329 (15)	0.0294 (13)	0.0031 (11)	−0.0023 (11)	0.0043 (11)
C20	0.060 (2)	0.0308 (15)	0.0440 (17)	0.0067 (14)	−0.0009 (15)	0.0087 (13)
C21	0.0573 (19)	0.0299 (14)	0.0412 (15)	−0.0087 (13)	0.0024 (13)	0.0082 (12)
C22	0.0418 (16)	0.0282 (13)	0.0335 (12)	−0.0061 (12)	0.0032 (11)	−0.0025 (10)
C23	0.0369 (16)	0.0483 (18)	0.0491 (18)	−0.0052 (13)	0.0065 (13)	0.0055 (15)
C24	0.0378 (18)	0.083 (3)	0.066 (2)	−0.0052 (18)	−0.0062 (16)	0.014 (2)
C25	0.0435 (17)	0.0484 (19)	0.0450 (17)	0.0009 (13)	−0.0045 (13)	0.0088 (14)
C26	0.066 (3)	0.106 (4)	0.061 (3)	−0.001 (2)	−0.012 (2)	0.010 (2)
B1	0.061 (2)	0.045 (2)	0.050 (2)	−0.0017 (17)	−0.0056 (17)	0.0075 (17)
F1	0.118 (3)	0.241 (5)	0.0664 (18)	−0.026 (3)	0.0212 (19)	−0.049 (3)

### Geometric parameters (Å, °)

**Table:**

Rh1—C2	2.209 (3)	C13—H13B	0.9700
Rh1—C6	2.211 (3)	C14—C17	1.528 (5)
Rh1—C1	2.251 (3)	C14—H14A	0.9800
Rh1—C5	2.262 (3)	C15—C16	1.526 (4)
Rh1—P2	2.2702 (8)	C15—H15A	0.9700
Rh1—P1	2.2754 (8)	C15—H15B	0.9700
P1—C9	1.839 (3)	C16—H16A	0.9600
P1—C11	1.850 (3)	C16—H16B	0.9600
P1—C14	1.869 (3)	C16—H16C	0.9600
P2—C10	1.839 (3)	C17—C18	1.524 (5)
P2—C19	1.840 (3)	C17—H17A	0.9700
P2—C22	1.877 (3)	C17—H17B	0.9700
C1—C2	1.366 (5)	C18—H18A	0.9600
C1—C8	1.514 (5)	C18—H18B	0.9600
C1—H1A	0.9800	C18—H18C	0.9600
C2—C3	1.503 (5)	C19—C20	1.523 (4)
C2—H2A	0.9800	C19—C25	1.536 (5)
C3—C4	1.531 (6)	C19—H19A	0.9800
C3—H3A	0.9700	C20—C21	1.524 (5)
C3—H3B	0.9700	C20—H20A	0.9700
C4—C5	1.511 (6)	C20—H20B	0.9700
C4—H4A	0.9700	C21—C22	1.537 (4)
C4—H4B	0.9700	C21—H21A	0.9700
C5—C6	1.363 (7)	C21—H21B	0.9700
C5—H5A	0.9800	C22—C23	1.517 (4)
C6—C7	1.506 (5)	C22—H22A	0.9800
C6—H6A	0.9800	C23—C24	1.524 (5)
C7—C8	1.516 (5)	C23—H23A	0.9700
C7—H7A	0.9700	C23—H23B	0.9700
C7—H7B	0.9700	C24—H24A	0.9600
C8—H8A	0.9700	C24—H24B	0.9600
C8—H8B	0.9700	C24—H24C	0.9600
C9—C10	1.525 (4)	C25—C26	1.491 (5)
C9—H9A	0.9700	C25—H25A	0.9700
C9—H9B	0.9700	C25—H25B	0.9700
C10—H10A	0.9700	C26—H26A	0.9600
C10—H10B	0.9700	C26—H26B	0.9600
C11—C15	1.526 (4)	C26—H26C	0.9600
C11—C12	1.531 (4)	B1—F1	1.362 (3)
C11—H11A	0.9800	B1—F2	1.372 (4)
C12—C13	1.518 (5)	B1—F4	1.377 (4)
C12—H12A	0.9700	B1—F3'	1.381 (4)
C12—H12B	0.9700	B1—F3	1.382 (4)
C13—C14	1.531 (4)	B1—F4'	1.383 (4)
C13—H13A	0.9700	B1—F2'	1.384 (4)
			
C2—Rh1—C6	95.24 (13)	C14—C13—H13A	109.6
C2—Rh1—C1	35.65 (13)	C12—C13—H13B	109.6
C6—Rh1—C1	80.79 (11)	C14—C13—H13B	109.6
C2—Rh1—C5	80.57 (12)	H13A—C13—H13B	108.2
C6—Rh1—C5	35.46 (17)	C17—C14—C13	112.7 (3)
C1—Rh1—C5	87.29 (11)	C17—C14—P1	115.5 (2)
C2—Rh1—P2	153.68 (10)	C13—C14—P1	105.2 (2)
C6—Rh1—P2	96.08 (9)	C17—C14—H14A	107.7
C1—Rh1—P2	170.65 (9)	C13—C14—H14A	107.7
C5—Rh1—P2	95.26 (9)	P1—C14—H14A	107.7
C2—Rh1—P1	97.05 (8)	C11—C15—C16	112.0 (3)
C6—Rh1—P1	151.59 (13)	C11—C15—H15A	109.2
C1—Rh1—P1	94.65 (8)	C16—C15—H15A	109.2
C5—Rh1—P1	172.93 (13)	C11—C15—H15B	109.2
P2—Rh1—P1	83.91 (2)	C16—C15—H15B	109.2
C9—P1—C11	105.88 (13)	H15A—C15—H15B	107.9
C9—P1—C14	103.23 (13)	C15—C16—H16A	109.5
C11—P1—C14	94.99 (12)	C15—C16—H16B	109.5
C9—P1—Rh1	109.13 (9)	H16A—C16—H16B	109.5
C11—P1—Rh1	124.17 (9)	C15—C16—H16C	109.5
C14—P1—Rh1	116.93 (10)	H16A—C16—H16C	109.5
C10—P2—C19	106.43 (13)	H16B—C16—H16C	109.5
C10—P2—C22	102.82 (13)	C18—C17—C14	112.0 (3)
C19—P2—C22	94.75 (13)	C18—C17—H17A	109.2
C10—P2—Rh1	109.28 (9)	C14—C17—H17A	109.2
C19—P2—Rh1	123.44 (10)	C18—C17—H17B	109.2
C22—P2—Rh1	117.61 (10)	C14—C17—H17B	109.2
C2—C1—C8	125.6 (3)	H17A—C17—H17B	107.9
C2—C1—Rh1	70.51 (16)	C17—C18—H18A	109.5
C8—C1—Rh1	110.2 (2)	C17—C18—H18B	109.5
C2—C1—H1A	114.1	H18A—C18—H18B	109.5
C8—C1—H1A	114.1	C17—C18—H18C	109.5
Rh1—C1—H1A	114.1	H18A—C18—H18C	109.5
C1—C2—C3	128.4 (3)	H18B—C18—H18C	109.5
C1—C2—Rh1	73.84 (17)	C20—C19—C25	115.7 (3)
C3—C2—Rh1	106.9 (2)	C20—C19—P2	103.7 (2)
C1—C2—H2A	113.2	C25—C19—P2	115.0 (2)
C3—C2—H2A	113.2	C20—C19—H19A	107.3
Rh1—C2—H2A	113.2	C25—C19—H19A	107.3
C2—C3—C4	113.2 (4)	P2—C19—H19A	107.3
C2—C3—H3A	108.9	C21—C20—C19	105.9 (3)
C4—C3—H3A	108.9	C21—C20—H20A	110.6
C2—C3—H3B	108.9	C19—C20—H20A	110.6
C4—C3—H3B	108.9	C21—C20—H20B	110.6
H3A—C3—H3B	107.7	C19—C20—H20B	110.6
C5—C4—C3	114.2 (3)	H20A—C20—H20B	108.7
C5—C4—H4A	108.7	C20—C21—C22	109.0 (3)
C3—C4—H4A	108.7	C20—C21—H21A	109.9
C5—C4—H4B	108.7	C22—C21—H21A	109.9
C3—C4—H4B	108.7	C20—C21—H21B	109.9
H4A—C4—H4B	107.6	C22—C21—H21B	109.9
C6—C5—C4	125.2 (4)	H21A—C21—H21B	108.3
C6—C5—Rh1	70.2 (2)	C23—C22—C21	112.1 (3)
C4—C5—Rh1	109.5 (2)	C23—C22—P2	117.0 (2)
C6—C5—H5A	114.4	C21—C22—P2	104.2 (2)
C4—C5—H5A	114.4	C23—C22—H22A	107.7
Rh1—C5—H5A	114.4	C21—C22—H22A	107.7
C5—C6—C7	127.3 (4)	P2—C22—H22A	107.7
C5—C6—Rh1	74.32 (19)	C22—C23—C24	114.8 (3)
C7—C6—Rh1	105.8 (2)	C22—C23—H23A	108.6
C5—C6—H6A	113.7	C24—C23—H23A	108.6
C7—C6—H6A	113.7	C22—C23—H23B	108.6
Rh1—C6—H6A	113.7	C24—C23—H23B	108.6
C6—C7—C8	115.2 (4)	H23A—C23—H23B	107.5
C6—C7—H7A	108.5	C23—C24—H24A	109.5
C8—C7—H7A	108.5	C23—C24—H24B	109.5
C6—C7—H7B	108.5	H24A—C24—H24B	109.5
C8—C7—H7B	108.5	C23—C24—H24C	109.5
H7A—C7—H7B	107.5	H24A—C24—H24C	109.5
C1—C8—C7	113.3 (3)	H24B—C24—H24C	109.5
C1—C8—H8A	108.9	C26—C25—C19	113.1 (3)
C7—C8—H8A	108.9	C26—C25—H25A	109.0
C1—C8—H8B	108.9	C19—C25—H25A	109.0
C7—C8—H8B	108.9	C26—C25—H25B	109.0
H8A—C8—H8B	107.7	C19—C25—H25B	109.0
C10—C9—P1	107.57 (19)	H25A—C25—H25B	107.8
C10—C9—H9A	110.2	C25—C26—H26A	109.5
P1—C9—H9A	110.2	C25—C26—H26B	109.5
C10—C9—H9B	110.2	H26A—C26—H26B	109.5
P1—C9—H9B	110.2	C25—C26—H26C	109.5
H9A—C9—H9B	108.5	H26A—C26—H26C	109.5
C9—C10—P2	107.82 (19)	H26B—C26—H26C	109.5
C9—C10—H10A	110.1	F1—B1—F2	119.1 (4)
P2—C10—H10A	110.1	F1—B1—F4	89.5 (5)
C9—C10—H10B	110.1	F2—B1—F4	114.3 (5)
P2—C10—H10B	110.1	F1—B1—F3'	99.5 (4)
H10A—C10—H10B	108.5	F2—B1—F3'	81.1 (5)
C15—C11—C12	115.7 (2)	F4—B1—F3'	155.6 (6)
C15—C11—P1	115.8 (2)	F1—B1—F3	106.4 (4)
C12—C11—P1	102.44 (19)	F2—B1—F3	109.9 (4)
C15—C11—H11A	107.5	F4—B1—F3	116.4 (5)
C12—C11—H11A	107.5	F1—B1—F4'	125.4 (4)
P1—C11—H11A	107.5	F2—B1—F4'	109.1 (4)
C13—C12—C11	107.6 (2)	F4—B1—F4'	46.1 (4)
C13—C12—H12A	110.2	F3'—B1—F4'	112.3 (5)
C11—C12—H12A	110.2	F3—B1—F4'	77.7 (4)
C13—C12—H12B	110.2	F1—B1—F2'	109.4 (4)
C11—C12—H12B	110.2	F4—B1—F2'	90.9 (5)
H12A—C12—H12B	108.5	F3'—B1—F2'	107.0 (5)
C12—C13—C14	110.1 (3)	F3—B1—F2'	134.7 (4)
C12—C13—H13A	109.6	F4'—B1—F2'	102.3 (4)
